# Supplementary material for: Nurses’ perspectives on using mobile health applications in southeastern Iran: Awareness, attitude, and obstacles
Source: PLoS One. 2025 Mar 10;20(3):e0316631. doi: 10.1371/journal.pone.0316631 (PMC11892810; doi:10.1371/journal.pone.0316631)
Supplement: S1 File — (PDF) [file pone.0316631.s002.pdf]

Zahedan University Of Medical Sciences

Research Ethics Committees Certificate

Approval ID:	IR.ZAUMS.REC.1401.133	Approval Date:	2022-06-26
Evaluated by:	Research Ethics Committees of Zahedan University Of Medical Sciences		
Status:	Approved		
Approval Statement:	The project was found to be in accordance to the ethical principles and the national norms and standards for conducting Medical Research in Iran.<br>Notice:<br>1. Although the proposal has been approved by the Biomedical Research Ethics Committee, meeting the professional and legal requirements is the sole responsibility of the PI and other project collaborators.<br>2. This certificate is reliant on the proposal/documents received by this committee on 2022-06-26. The committee must be notified by the PI as soon as the proposal/documents are modified.		
Proposal Title:	Study of nurses' knowledge and attitude towards using mobile health applications in teaching hospitals of Zahedan University of Medical Sciences in 2022		
Principal Investigator:	Name: afsaneh karimi<br>Email: karimi.f@zaums.ac.ir		

Dr. Habib Ghaznavi
Committee Director
Zahedan University Of Medical Sciences

Dr. Noor Mohammad Bakhshani
Committee Secretary
Zahedan University Of Medical Sciences
