# Supplementary material for: Nurses’ perspectives on using mobile health applications in southeastern Iran: Awareness, attitude, and obstacles
Source: PLoS One. 2025 Mar 10;20(3):e0316631. doi: 10.1371/journal.pone.0316631 (PMC11892810; doi:10.1371/journal.pone.0316631)
Supplement: Attachment — Submitted filename: Response to Reviewers.docx [file pone.0316631.s003.docx]

**Dear Academic Editor and Reviewers,**

Thank you very much for your consideration, and we really appreciate the comments and have learned a lot. Appropriate changes were made in the revised manuscript according to the suggestions of reviewers and editor.

**Responses to academic editor comments:**

Financial disclosure:

- Please indicate whether the sponsors or funders play any role in the study design, data collection and analysis, decision to publish, or preparation of the manuscript?
- **Answer**: We appreciate your scientific and constructive comments. It was done accordingly in the financial disclosure section.

Ethical approval:

- Please add ethical approval as supporting document and remove the URL from methods section.
- **Answer**: It was done accordingly.

Methods:

- Study population and sample: please provide inclusion/exclusion criteria for the participants
- **Answer**: Inclusion criteria for participants were added in the method section. Page 5, lines 135-7
- Data collection tool was explained in detail. However, data collection process was not explained. Please provide explanations about how the data was collected (paper-base or electronically), who collected the data (interviewer or self-administered), how was the data transmitted, stored and cleaned e.t.c.
- **Answer**: data collection process was added accordingly. Page 5, lines 139-156

Results:

- Age group: please what was the basis for this categorization. The age intervals are not consistent across the age groups. Why and what is the rational for this. If there is any justification, please explain in the methods section otherwise use conventional age categorization with regular intervals.
- **Answer**: Despite the change and modification of the age categories and the recalculation of the relevant descriptive statistics (number and percentage) remained constant. Page 7, table 1.

Discussion:

- Study limitations were provided. Any strengths for the study?
- **Answer**: Strengths of the study were added. Page 12, lines 289-293.

**Journal Requirements:**

1. When submitting your revision, we need you to address these additional requirements.

Please ensure that your manuscript meets PLOS ONE's style requirements, including those for file naming. The PLOS ONE style templates can be found at 
<https://journals.plos.org/plosone/s/file?id=wjVg/PLOSOne_formatting_sample_main_body.pdf> and 
<https://journals.plos.org/plosone/s/file?id=ba62/PLOSOne_formatting_sample_title_authors_affiliations.pdf>

**Answer**: We reviewed the manuscript and made the necessary corrections.

2. Thank you for stating in your Funding Statement: 
This study was supported by the Vice Chancellor for Research and Technology of Zahedan University of Medical Sciences (No: 10494).

**Answer**: Thank you.

Please provide an amended statement that declares *all* the funding or sources of support (whether external or internal to your organization) received during this study, as detailed online in our guide for authors at <http://journals.plos.org/plosone/s/submit-now>.  Please also include the statement “There was no additional external funding received for this study.” in your updated Funding Statement. 
Please include your amended Funding Statement within your cover letter. We will change the online submission form on your behalf.

**Answer:** Done accordingly.

3. Thank you for stating the following in the Acknowledgments Section of your manuscript: 
The authors thank the Vice Chancellor for Research and Technology of Zahedan University of Medical Sciences for supporting the project. They also thankful to the nurses who participated in this study for sharing their valuable experiences.

We note that you have provided funding information that is not currently declared in your Funding Statement. However, funding information should not appear in the Acknowledgments section or other areas of your manuscript. We will only publish funding information present in the Funding Statement section of the online submission form. 
Please remove any funding-related text from the manuscript and let us know how you would like to update your Funding Statement. Currently, your Funding Statement reads as follows: 
This study was supported by the Vice Chancellor for Research and Technology of Zahedan University of Medical Sciences (No: 10494).

Please include your amended statements within your cover letter; we will change the online submission form on your behalf.

**Answer:** Done accordingly.

4. We note that your Data Availability Statement is currently as follows: All relevant data are within the manuscript and its Supporting Information files.

Please confirm at this time whether or not your submission contains all raw data required to replicate the results of your study. Authors must share the “minimal data set” for their submission. PLOS defines the minimal data set to consist of the data required to replicate all study findings reported in the article, as well as related metadata and methods (<https://journals.plos.org/plosone/s/data-availability#loc-minimal-data-set-definition>).

For example, authors should submit the following data:

- The values behind the means, standard deviations and other measures reported;
- The values used to build graphs;
- The points extracted from images for analysis.

Authors do not need to submit their entire data set if only a portion of the data was used in the reported study.

If your submission does not contain these data, please either upload them as Supporting Information files or deposit them to a stable, public repository and provide us with the relevant URLs, DOIs, or accession numbers. For a list of recommended repositories, please see <https://journals.plos.org/plosone/s/recommended-repositories>.

If there are ethical or legal restrictions on sharing a de-identified data set, please explain them in detail (e.g., data contain potentially sensitive information, data are owned by a third-party organization, etc.) and who has imposed them (e.g., an ethics committee). Please also provide contact information for a data access committee, ethics committee, or other institutional body to which data requests may be sent. If data are owned by a third party, please indicate how others may request data access.

**Answer**: We apologize for the unwanted ambiguity. We uploaded Supporting information files.

5. When completing the data availability statement of the submission form, you indicated that you will make your data available on acceptance. We strongly recommend all authors decide on a data sharing plan before acceptance, as the process can be lengthy and hold up publication timelines. Please note that, though access restrictions are acceptable now, your entire data will need to be made freely accessible if your manuscript is accepted for publication. This policy applies to all data except where public deposition would breach compliance with the protocol approved by your research ethics board. If you are unable to adhere to our open data policy, please kindly revise your statement to explain your reasoning and we will seek the editor's input on an exemption. Please be assured that, once you have provided your new statement, the assessment of your exemption will not hold up the peer review process.

**Answer**: Thanks for your advice. We have corrected this section to adhere to your open data policy.

6. Please amend either the abstract on the online submission form (via Edit Submission) or the abstract in the manuscript so that they are identical.

**Answer:** Done accordingly.

7. Your ethics statement should only appear in the Methods section of your manuscript. If your ethics statement is written in any section besides the Methods, please delete it from any other section.

**Answer:** Done accordingly. Page 5, lines 126-9.

8. Please review your reference list to ensure that it is complete and correct. If you have cited papers that have been retracted, please include the rationale for doing so in the manuscript text, or remove these references and replace them with relevant current references. Any changes to the reference list should be mentioned in the rebuttal letter that accompanies your revised manuscript. If you need to cite a retracted article, indicate the article’s retracted status in the References list and also include a citation and full reference for the retraction notice.

**Answer:** Based on our review, the references were not a problem.

**Responses to Reviewers’ comments**

**Reviewer #1:** I consider the abstract to be technically sound and even more as a Nigerian, living in this time of coming of age of technology and given recent boom in mHealth technologies in developing nations, I consider it even more timely and apt. As a leader in the health informatics field, developing some of the mHealth innovations, I often stop to consider the impact of the many mHealth tools and solutions we churn out every now and then, targeting either the clinician or the patient. My understanding is that the author who I have worked with in the past, have decided to do more fundamental study to figure out this question around 'impact' and 'effectiveness'.
The studies have shown with clear and yet rigorous statistical analysis, following sound research methodology, what the answer to the question is by identifying critical variables of awareness, attitude, and obstacles and measuring their mean scores. Which anyone can attempt to reproduce if they wish. The Manuscript is easy to understand in first reading because of the authors have deployed intelligible straight-forward English narratives in their writing.

**Answer**: We are grateful to the reviewer#1 for your appreciation of the value of our work.

**Reviewer #2:** The manuscript raises important problem of mobile applications suitability for nurses and medical workers
Overall, information is too limited. It is restricted to the study at one city. It should be generalized to whole region, to developing countries.
The presentation material itself is too limited. The manuscript has only tables, no figures.
One large figure – scheme of experiment – will improve the material presentation

**Answer**: We appreciate the reviewer' valuable comments and constructive suggestions, which help improve the quality of the manuscript.

In the Abstract:
mHealth Apps – should be commented. It is any mobile application, in general, or program by some manufacturer? “Android operating system installed (82%)”

**Answer**: We are sorry for the unwanted ambiguity. We meant it in general. But the results of the study showed that most nurses use the Android operating system on their devices. We modified the title of the manuscript as well as the introduction subsection of the abstract section for clarity. Page 2, lines 33-35.

Don’t use passive voice in English (“were used”, “was done”). Write directly.

**Answer**: We appreciate your scientific comment and suggestion. We revised the sentences in the abstract and methods sections and used active voice sentences instead of passive ones.

Results section shows rather common statistics. Conclusion is not complete.

**Answer**: We agree with the reviewer. We revised the conclusion section to clear the ambiguity. Page 2, lines 49-56.

“Mobile Health”, “Obstacle” are not appropriate as keywords.

**Answer:** Both keywords “Mobile Health”and mHealth have been provided as entry terms of telemedicine the search for the keyword mHealth in Mesh Browser. Therefore, we added telemedicine to keywords. In addition, we removed the obstacle from the keywords section. If the reviewer does not agree with the keywords provided by us, we would be grateful if the reviewer would guide us in determining the keywords. Page 2, line 59.

The term “the digital health literacy” should be commented. It is kind of science jargon,

**Answer:** We agree with the reviewer. For this reason, we added a scientific and comprehensive definition in the discussion section to clear the ambiguity. Page 12, lines 269-72.

In the text “The estimate made in 2018…” - old data.

Answer: Thank you very much for the completely scientific comment of the reviewer. The indicated sentence was amended with a new sentence and date. Page 3, lines 68-9.

The term ‘mHealth’ refers to the paper in Persian. Need cite available literature, add references.

**Answer**: We agree with the reviewer and we added related references accordingly. Page 3, lines 70-72.

Please not use bulk citations – 3 or more references together, like “(15, 18-20)”, “(21-23)”. Separate phrases into parts, add detail.

**Answer:** We agree with the reviewer. we corrected all the cases that had conditions similar to those mentioned by the reviewer throughout the manuscript. Pages 3 and 4, lines 64-110.

Tables 3 and 4 – the results should be highlighted, show most interesting result, discuss it. Maybe present it in other visual form (like a histogram, heatmap).

**Answer**: According to the reviewer suggestions, most interesting result highlighted in the tables.

We agree with the reviewer that the results are better in the form of a combination of tables and visual format (especially in graphs form) in the articles. However, in our article, Tables 3 and 4 contain the relevant details (Likert scale) and present the mean scores along with the relevant standard deviation, which cannot be expressed if the data is presented using visual format. Therefore, with respect to the reviewer's comment, we believe that presenting the data in the current format is better.

Conclusion should have general interest. Phrase like “The majority of nurses use smartphones (86%) and Android operating systems (82%)” gives no information. Nurses where?

**Answer**: we agree with the reviewer and apologize for any unintended ambiguity. we have revised the conclusion section to enhance clarity. Page 13, lines 301-12.

**Reviewer #3:** Some Concerns:

1. The authors indicated in the limitation section that the study was conducted in one city in the southeast of Iran therefore the results cannot be generalized to the whole country not to talk of several developing countries. So, the geographical coverage, sample size and the findings in this paper does not aligning with title of the paper. Therefore, the author should consider narrowing the title to reflect the locations/region where the study was conducted. Example “Nurses' Perspectives on Using mHealth Apps in Southeastern Iran: Awareness, Attitude, and Obstacles”.

**Answer:** Thank you for the constructive comments to our manuscript. We agree with the reviewer comment and suggestion. We modified the title accordingly as follows. **Page 1, lines 1-2.**

"Nurses' perspectives on using mHealth apps in southeastern Iran: Awareness, attitude, and obstacles"

2. The purpose of the discussion section is to interpret results and justify conclusion. The discussion section seems to be too long, citing lots of works done in other countries, I suggest that some of those literatures should be moved to introduction section where past related works were quoted. You need to restate your key results in this section.

**Answer:** We agree with the reviewer suggestion. We moved two literatures from the discussion to the introduction section. In addition, we removed two less relevant literature items from the discussion section. Page 4, lines 109-11.
